# Using a Vibrotactile Biofeedback Device to Augment Foot Pressure During Walking in Healthy Older Adults: A Brief Report

**DOI:** 10.3389/fpsyg.2019.01008

**Published:** 2019-05-08

**Authors:** Kazuhiro Yasuda, Yuki Hayashi, Anna Tawara, Hiroyasu Iwata

**Affiliations:** ^1^Research Institute for Science and Engineering, Waseda University, Tokyo, Japan; ^2^Graduate School of Creative Science and Engineering, Waseda University, Tokyo, Japan; ^3^Department of Modern Mechanical Engineering, Waseda University, Tokyo, Japan

**Keywords:** older, sensory augmentation, human-machine interface, gait training, dual task

## Abstract

Human movement based on sensory control is significant to motor task performance. Thus, impairments to sensory input significantly limit feedback-type motor control. The present study introduces a vibrotactile biofeedback (BF) system which augments information regarding the user’s foot pressure to enhance gait performance. The effects of the proposed system on the gait patterns of healthy older adults and on the cognitive load during gait were evaluated; these factors are essential to clarify feasibility of the device in real-life settings. The primary task of our study was to evaluate gait along with a cognitively demanding activity in 10 healthy older adults. Regarding kinematic and kinetic data in the BF condition, the subjects had significantly increased ankle dorsiflexion during the heel contact phase in the sagittal plane and marginally increased foot pressure at the toe-off and stride length. However, such kinematic and kinetic changes were not attributed to the increased walking speed. In addition, cognitive performance (i.e., the number of correct answers) was significantly decreased in participants during gait measurements in the BF condition. These data suggest that the system had the potential for modifying the kinematic and kinetic patterns during walking but not the more comprehensive walking performance in older adults. Moreover, the device appears to place a cognitive load on older adults. This short report provides crucial primary data that would help in designing successful sensory augmentation devices and further research on a BF system.

## Introduction

Gait performance is important to independently perform activities of daily living, and it is an important measure of functional capacity among the elderly people (Fritz and Lusardi, 2009). Moreover, gait performance can help predict adverse events (Montero-Odasso et al., 2005), disability, and mortality in older adults (Studenski et al., 2011). Aging negatively affects spatiotemporal gait parameters, such as shorter stride length, wider base of support, variability gait duration, and slower walking speed (Aboutorabi et al., 2016). Aging-related variations in gait parameters are associated with body structure and cognitive function changes (Tian et al., 2017). Therefore, preserving gait performance is important for older adult health and fall prevention (Fritz and Lusardi, 2009).

Sensory augmentation is a technique to enhance or supplement sensory information to improve information processing and performance (Bach-y-Rita et al., 1969). This type of technique provides specific sensory feedback (e.g., visual, auditory, or tactile feedback) of body fluctuation or gait patterns during training. Compared with other methods, rhythmic auditory stimulation (RAS) was found beneficial in enhancing the gait performance of patients with Parkinson’s disease (PD) (Thaut et al., 1996; Pau et al., 2016); associated studies have shown that artificial RAS (i.e., metronome or music-sounds) and ecological RAS (i.e., personalized actual footstep sounds) are equally effective in improving gait performance in PD (Murgia et al., 2018). Recently, a multisensory approach established on action observation plus sonification (i.e., auditory feedback acquired by transforming kinematic data of the movements of relevant body parts) was reported to help patients with PD with freezing gait to relearn gait movements (Mezzarobba et al., 2018). Various methods have been established to provide biofeedback (BF) on body fluctuation to older adults or patients with neurological disorders during stance (Dozza et al., 2007) or gait (Verhoeff et al., 2009; Davis et al., 2010) tasks. Moreover, most previous reports have emphasized on the head or trunk positions as representative body movements (Dozza et al., 2007; Horak et al., 2009; Verhoeff et al., 2009; Davis et al., 2010; Wall, 2010). However, proper foot movement is important to progress with the supporting foot during stance. In particular, appropriate heel strike and push off during walking play an important role in the attenuation of impact forces, and assist in forward propulsion (Whittle, 1999). Further, previous studies have reported that compared with young adults, older adults have reduced ankle dorsiflexion at initial contact and reduced plantarflexion at toe-off (Judge et al., 1996; Kerrigan et al., 1998; Arnold et al., 2014). These studies have demonstrated the importance of appropriate foot motion for the maintenance and improvement of gait performance.

It has been recently shown that vibrotactile feedback (VTF) can influence postural and gait performance during dual tasks and cognitive task performance (Verhoeff et al., 2009; Haggerty et al., 2012). This may be attributable to the fact that VTF necessitates participants to engage in higher cognitive processes to deal with the stimulus. In particular, the elderly would be vulnerable to decreased dual-task performance during gait tasks or postural control (Woollacott and Shumway-Cook, 2002; Fraizer and Mitra, 2008). Therefore, such an increase in cognitive load should be taken into account in the application of VTF to real-life situations. In this study, we introduced a vibrotactile BF system that can provide information on the foot pressure pattern to improve optimal foot movements (Judge et al., 1996; Kerrigan et al., 1998; Whittle, 1999; Arnold et al., 2014). First, we examined the influence of the proposed BF system on the gait pattern of 10 healthy older adults in an initial validity study. Thereafter, we aimed to clarify the influence of the developed BF device on cognitive burden during gait because this aspect is necessary to clarify its feasibility in real-life settings.

## Materials and Methods

### System Overview

**FIGURE 1:**
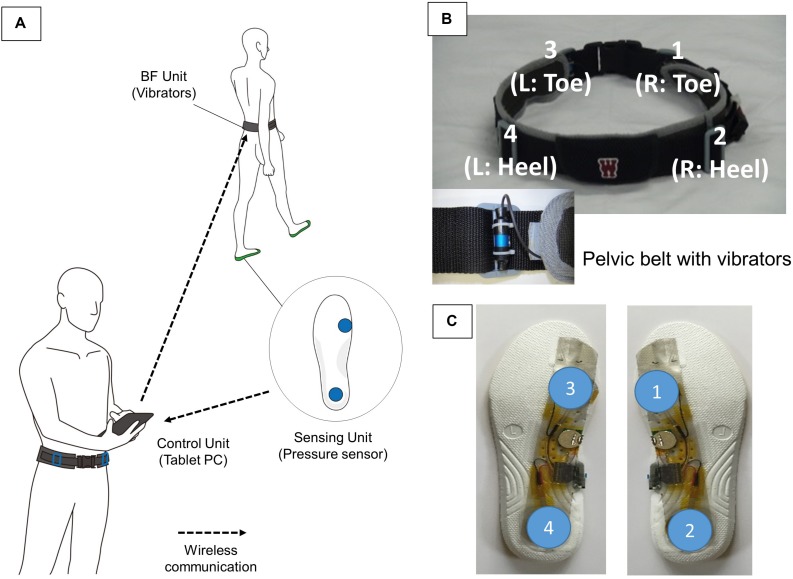
System overview. **(A)** Our devices consist of a shoe insole with a foot pressure sensor, vibrotactile BF device (pelvic belt), and personal computer. The system augments the foot pressure pattern by using the vibratory belt attached to the pelvis. By using the feedback information, our system provides the user with accurate information regarding their gait pattern. **(B)** Vibrators are placed on the anterior and posterior-superior iliac spines to easily understand the tactile stimuli. During gait training, the vibrators on the trainee’s pelvic belts are simultaneously activated corresponding to the user’s foot presser sensor. **(C)** The sensing unit includes two built-in pressure sensors, and the sensor placement is designed to easily understand heel strike and push off. Using this information from insole sensors, the user can be aware of the timing, and intensity of their heel strike and push off.

The biofeedback device consists of a BF unit (four vibrators in the belt), a sensing unit (foot pressure sensor), and a personal computer (PC) (Figure 1A). The system was designed for transmitting the timing and strength of heel contact and push off, since ankle dorsiflexion at heel contact, and push off at heel off are important foot motions during walking in older adults (Judge et al., 1996; Kerrigan et al., 1998; Whittle, 1999; Arnold et al., 2014).

Two built-in pressure sensors are found in the sensing unit, and sensor placement is designed to easily understand the timing of heel strike and push off (Figure 1C). The proposed system enhances the foot pressure pattern with four vibrators attached to the pelvis. The pelvis was chosen because (a) the anterior-superior iliac spine and posterior-superior iliac spine are large osteophytes and readily detect vibration and (b) stimulation patterns are easy to understand as anterior and posterior parts of the pelvis and anterior and posterior parts of the foot (toe and heel) are in the same horizontal planes, respectively. Four vibrators (Figure 1B) in the pelvic belt facilitate the older adult participant to convey foot contact information (i.e., timing and intensity of foot pressure). A vibration at a frequency of 80 Hz was applied to the pelvis in the stance phase in synchronization with the earth connection of the foot pressure sensor. The small number of vibrators helps the users easily understand BF input. The importance of the vibration is comparative to that in the foot pressure sensors in the shoes.

By using the proposed feedback system, our device provides accurate information regarding the trainees’ gait pattern. In gait training, the older adults are provided BF to modify the gait patterns properly. The foot pressure sensor should correctly obtain pressure data during gait and send data back to the PC.

### Participants

This study included 10 healthy older adults (5 males and 5 females, mean age; 71.9 ± 2.6 years). Participants were recruited from the Shinjuku-ward, Tokyo, which was facilitated through local advertising by the Human Resources Center in Shinjuku-ward. At the initial visit, screening physical and hearing examination were performed. The inclusion criteria were as follows: age ≥65 years, sufficient communication skills to understand the instructions, living independently in the community, able to walk without an aid, free of neurological or musculoskeletal disorders that might influence gait performance or cognition (stroke, brain trauma, PD, acute illness, and significant orthopedic disability), mini-mental state examination score >20 (no dementia), and ability to sense vibrations of the BF system (during screening evaluation, vibration was actually applied to the pelvis to confirm reactions). Furthermore, participants were excluded if they had a hearing deficit, nerve damage, body pain, severe visual impairment, a history of fainting, or a body mass index >30 kg/m^2^.

### Ethics Approval and Consent to Participate and Publication

Procedures in this study were approved by the Waseda University Ethics Committee for Human Research. After a complete description of the procedures and purpose of the study, written informed consent was obtained from all participants.

### Procedure

In the validation study, the main task was gait and a cognitive-demanding task (i.e., serial subtraction task) (Ellmers et al., 2016). Participants walked for 1 min along the corridor at the same time performed a serial subtraction task. Before the 1-min walking task, each participant sat down on a chair and received an adequate explanation from the experimenter regarding the relationship between the sensor and the vibrator. Next, participants practiced a 10-m walking task to better understand the relationship between the foot pressure sensor and the vibration.

During the walking task, participants counted backward aloud in increments of seven from a starting number. The initial number was determined from 125 to 250 (Ellmers et al., 2016). Then, for the gait session, a different number was randomly selected while subtracting. Further, in the BF condition, the older adults walked and corrected the gait pattern with BF information while attempting the serial subtracting task. In the control (No-BF) condition, the older adults only walked while subtracting.

Biofeedback conditions and control conditions were measured at a 1-week interval in counterbalanced order among participants for avoiding potential order effect. All measurements associated with walking and cognition tasks in this study were performed by part-time research assistants blinded to the BF or no-BF condition.

### Measurements and Analysis

The following were measurements used to examine the participants’ walking performance: (a) ankle dorsiflexion at heel strike as kinematic data, (b) maximum foot pressure at the push off as kinetic data, (c) stride length as a measure of performance of walking, and (d) walking speed as the comprehensive evaluation of walking. Inertial sensors was used to measure kinematic data during walking (Rehagait, Hasomed, Germany). During the 1 minute of walking, acceleration, and deceleration data for motion capture in the walking test were excluded from the analyses. The maximum foot pressure at push off, stride length during walking, and walking speed were measured using a force plate (P-walk, BTS, Italy). Data were acquired with a 2-m long reaction force sensor placed 5 m away from the starting point for walking. For data (a), (b), and (c), mean values of the left and right leg were used.

The cognitive performance score was the number of responses and correct verbalized arithmetic calculations (Ellmers et al., 2016). During trials, dual-task scores were calculated when the older adults simultaneously performed the walking and cognitive tasks. The number of responses and correct arithmetic calculations verbalized by participants were calculated for the BF condition and control condition.

Data normality was evaluated with the Shapiro – Wilk test, and a non-parametric test was used if a violation of normality was noted. With respect to ankle dorsiflexion in the sagittal plane, maximum foot pressure at push off, and stride length, multiple *t*-tests with correction for multiple comparison using the Holm–Sidak method were applied using GraphPad Prism software (Streiner, 2015) (Graphpad Prism version 6.0, GraphPad Software Inc., CA, United States). Differences were considered to be significant at *p* < 0.05. As for walking speed and cognitive performance, Student’s *t*-test or the Wilcoxon signed-rank test was used for comparing the BF and control conditions with a *p*-value of <0.05 considered to be statistically significant.

## Results

Table 1 presents the results of walking and cognitive performance of older adults during the experiment. With respect to ankle dorsiflexion in the sagittal plane, the participants in the BF condition had increased ankle dorsiflexion angle at the heel contact phase than in the control condition [*t*(18) = 2.412, *p* = 0.027]. In the BF condition, participants had marginally higher foot pressure at toe off than the controls [*t*(18) = 1.956, *p* = 0.066]. Participants using BF marginally extended their stride length [*t*(18) = 2.072, *p* = 0.053] compared with the control. However, there were no significant difference between BF and the control conditions for walking speed [*t*(9) = 1.462, *p* = 0.177].

**TABLE 1:** Variables for walking and cognitive performance during the experiment.

Walking performance (*n* = 10)			
	BF	Control (No-BF)	*p-*value
Ankle joint angle (degrees)	25.3 ± 1.4	20.1 ± 5.1	0.027*
Foot pressure (hpa)	80.6 ± 4.2	77.1 ± 3.6	0.066^†^
Stride length (m)	1.2 ± 0.1	1.1 ± 0.1	0.053^†^
Walking speed (m/s)	1.0 ± 0.2	1.0 ± 0.1	0.177
**Cognitive performance (*n* = 10)**
Number of response	12.1 ± 5.1	12.5 ± 5.6	0.373
Correct answers	8.0 ± 4.8	9.9 ± 5.7	0.014*

Values are denoted in mean ± SD. BF, biofeedback; ^†^*p* < 0.1, *p < 0.05.

Regarding cognitive performance, no significant difference was observed in the number of answers between BF and control conditions [*t*(9) = 0.937, *p* = 0.373]. The number of correct arithmetic calculations was decreased in the participants during gait in the BF condition [*t*(9) = 3.031, *p* = 0.014] compared with the control condition.

## Discussion

A proposed device that augments foot pressure information was introduced in this study. The validation study explores the feasibility (i.e., effects of gait performance and cognitive burden) of the BF system in 10 older participants. In walking ability, the BF system helped participants increase their ankle dorsiflexion angle in the sagittal plane at the heel contact phase and marginally increased foot pressure at the toe-off and stride length. However, these kinematic and kinetic changes have no influence on the increased walking speed required for comprehensive walking performance. Furthermore, cognitive performance (i.e., the number of correct arithmetic calculations) was significantly decreased in the BF condition. Therefore, although there are beneficial kinematic and kinetic changes, the proposed BF system may need higher information awareness from the BF of the participants.

Appropriate foot movements contribute to shock absorption and allow the body to move forward with the supporting foot during walking (Aboutorabi et al., 2016). Proposed BF system may induce positive kinematic and kinetic changes in field trials, as the BF information directly conveys the characteristics of the foot contact pattern. However, these changes did not result in improvements in walking speed. A previous study showed that older adults tend to adopt a conservative gait pattern when they walk in an unfamiliar environment (Menz et al., 2003). This tendency may explain why walking speed did not change under the BF condition in the present study. For addressing this limitation, the effect of habituation after repeated practice must be assessed.

Furthermore, it is necessary to address the points to be improved. Our device only used four vibrators to reduce the cognitive burden during BF training. Nevertheless, the older participants demonstrated a decrease in cognitive performance during gait tasks. This is probably due to the low working memory in older adults (Woollacott and Shumway-Cook, 2002; Fraizer and Mitra, 2008; Lovden et al., 2008). As mentioned in the introduction, previous reports have suggested that VTF can affect gait performance during dual tasks and affect cognitivetask performance (Verhoeff et al., 2009). This may be because VTF requires older adults to perform higher cognitive processes to deal with the information from the BF device. Given that the older adults are susceptible to decreased cognitive performance during gait tasks, the increase in cognitive load with BF device should be considered when attempting to apply VTF.

This feasibility study was performed to examine the feasibility of the proposed BF system. However, the sample size was relatively small. Thus, a more rigorous study with a larger sample size is required. Moreover, future studies should include young participants to clarify the effect of BF gait training on cognitive and motor performance.

Thus, the BF system may have the potential to modify the kinematic and kinetic patterns, but not the walking speed (i.e., comprehensive walking performance) in older adults. Moreover, it is likely that the even four vibratory stimuli placed an increased cognitive load, which could be linked to the limited capacity of working memory in older people. In future trials, this aspect should be considered to establish a cutoff value for age or improve the proposed device. This report provides essential initial data for successfully designing sensory augmentation devices and exploring future academic issues.

## Ethics Statement

All procedureswere approved by the Waseda University Ethics Committee for Human Research. After a complete description of the procedures and purpose of the study, written informed consent was obtained from all participants.

## Author Contributions

KY designed the study, collected and analyzed the data, and drafted the manuscript. YH and AT made contributions toward data development and analyses. HI was involved in the conception of the system. All authors agreed with the final contents of the manuscript.

## Conflict of Interest Statement

The authors declare that the research was conducted in the absence of any commercial or financial relationships that could be construed as a potential conflict of interest.

## References

[B1] Aboutorabi A. Arazpour M. Bahramizadeh M. Hutchins S. W. Fadayevatan R. (2016). The effect of aging on gait parameters in able-bodied older subjects: a literature review. *Aging Clin. Exp. Res.* 28 393–405. 10.1007/s40520-015-0420-6 26210370

[B2] Arnold J. B. Mackintosh S. Jones S. Thewlis D. (2014). Differences in foot kinematics between young and older adults during walking. *Gait Posture* 39 689–694. 10.1016/j.gaitpost.2013.09.021 24183676

[B3] Bach-y-Rita P. Collins C. C. Saunders F. A. White B. Scadden L. (1969). Vision substitution by tactile image projection. *Nature* 221 963–964. 10.1038/221963a0 5818337

[B4] Davis J. R. Carpenter M. G. Tschanz R. Meyes S. Debrunner D. Burger J. (2010). Trunk sway reductions in young and older adults using multi-modal biofeedback. *Gait Posture* 31 465–472. 10.1016/j.gaitpost.2010.02.002 20206528

[B5] Dozza M. Horak F. B. Chiari L. (2007). Auditory biofeedback substitutes for loss of sensory information in maintaining stance. *Exp. Brain Res.* 178 37–48. 10.1007/s00221-006-0709-y 17021893

[B6] Ellmers T. J. Cocks A. J. Doumas M. Williams A. M. Young W. R. (2016). Gazing into thin air: The dual-task costs of movement planning and execution during adaptive gait. *PLoS One* 11:e0166063. 10.1371/journal.pone.0166063 27824937 PMC5100909

[B7] Fraizer E. V. Mitra S. (2008). Methodological and interpretive issues in posture-cognition dual-tasking in upright stance. *Gait Posture* 27 271–279. 10.1016/j.gaitpost.2007.04.002 17524648

[B8] Fritz S. Lusardi M. (2009). White paper: “walking speed: the sixth vital sign”. *J. Geriatr. Phys. Ther.* 32 46–49. 20039582

[B9] Haggerty S. Jiang L. T. Galecki A. Sienko K. H. (2012). Effects of biofeedback on secondary-task response time and postural stability in older adults. *Gait Posture* 35 523–528. 10.1016/j.gaitpost.2011.10.359 22406291 PMC3772646

[B10] Horak F. B. Dozza M. Peterka R. Chiari L. Wall I. I. I. C. (2009). Vibrotactile biofeedback improves tandem gait in patients with unilateral vestibular loss. *Ann. N.Y. Acad. Sci.* 1164 279–281. 10.1111/j.1749-6632.2008.03707.x 19645912 PMC2721155

[B11] Judge J. O. Davis R. B. III Ounpuu S. (1996). Step length reductions in advanced age: the role of ankle and hip kinetics. *J. Gerontol. A Biol. Sci. Med. Sci.* 51 M303–M312. 8914503 10.1093/gerona/51a.6.m303

[B12] Kerrigan D. C. Todd M. K. Della Croce U. Lipsitz L. A. Collins J. J. (1998). Biomechanicalgait alterations independent of speed in the healthy elderly: evidence forspecific limiting impairments. *Arch. Phys. Med. Rehabil.* 79 317–322. 10.1016/s0003-9993(98)90013-2 9523785

[B13] Lovden M. Schaefer S. Pohlmeyer A. E. Lindenberger U. (2008). Walking variability and working-memory load in aging: a dual-process account relating cognitive control to motor control performance. *J. Gerontol. B Psychol. Sci. Soc. Sci.* 63 121–128. 18559676 10.1093/geronb/63.3.p121

[B14] Menz H. B. Lord S. R. Fitzpatrick R. C. (2003). Age-related differences in walkingstability. *Age Ageing* 32 137–142. 12615555 10.1093/ageing/32.2.137

[B15] Mezzarobba S. Grassi M. Pellegrini L. Catalan M. Kruger B. Furlanis G. (2018). Action observation plus sonification. a novel therapeutic protocol for parkinson’s patient with freezing of Gait. *Front. Neurol.* 8:723. 10.3389/fneur.2017.00723 29354092 PMC5758544

[B16] Montero-Odasso M. Schapira M. Soriano E. R. Varela M. Kaplan R. Camera L. A. (2005). Gait velocity as a single predictor of adverse events in healthy seniors aged 75 years and older. *J. Gerontol. A Biol. Sci. Med. Sci.* 60 1304–1309. 10.1093/gerona/60.10.1304 16282564

[B17] Murgia M. Pili R. Corona F. Sors F. Agostini T. A. Bernardis P. (2018). The use of footstep sounds as rhythmic auditory stimulation for gait rehabilitation in parkinson’s disease: a randomized controlled trial. *Front. Neurol.* 9:348. 10.3389/fneur.2018.00348 29910764 PMC5992388

[B18] Pau M. Corona F. Pili R. Casula C. Sors F. Agostini T. (2016). Effects of physical rehabilitation integrated with rhythmic auditory stimulation on spatio-temporal and kinematic parameters of gait in parkinson’s disease. *Front. Neurol.* 7:126 10.3389/fneur.2016.00126 PMC4980587 27563296

[B19] Streiner D. L. (2015). Best (but oft-forgotten) practices: the multiple problems of multiplicity-whether and how to correct for many statistical tests. *Am. J. Clin. Nutr.* 102 721–728. 10.3945/ajcn.115.113548 26245806

[B20] Studenski S. Perera S. Patel K. Rosano C. Faulkner K. Inzitari M. (2011). Gait speed and survival in older adults. *JAMA* 305 50–58. 10.1001/jama.2010.1923 21205966 PMC3080184

[B21] Thaut M. H. McIntosh G. C. Rice R. R. Miller R. A. Rathbun J. Brault J. M. (1996). Rhythmic auditory stimulation in gait training for Parkinson’s disease patients. *Mov. Disord.* 11 193–200. 10.1002/mds.870110213 8684391

[B22] Tian Q. Chastan N. Bair W. N. Resnick S. M. Ferrucci L. Studenski S. A. (2017). The brain map of gait variability in aging, cognitive impairment and dementia-A systematic review. *Neurosci. Biobehav. Rev.* 74 149–162. 10.1016/j.neubiorev.2017.01.020 28115194 PMC5303129

[B23] Verhoeff L. L. Horlings C. G. Janssen L. J. Bridenbaugh S. A. Allum J. H. (2009). Effects of biofeedback on trunk sway during dual tasking in the healthy young and elderly. *Gait Posture* 30 76–81. 10.1016/j.gaitpost.2009.03.002 19356934

[B24] Wall I. I. I. C. (2010). Application of vibrotactile feedback of body motion to improve rehabilitation in individuals with imbalance. *J. Neurol. Phys. Ther.* 34 98–104. 10.1097/NPT.0b013e3181dde6f0 20588096 PMC2898155

[B25] Whittle M. W. (1999). Generation and attenuation of transient impulsive forces beneath the foot: a review. *Gait Posture* 10 264–275. 10.1016/s0966-6362(99)00041-7 10567759

[B26] Woollacott M. Shumway-Cook A. (2002). Attention and the control of posture and gait: a review of an emerging area of research. *Gait Posture* 16 1–14. 10.1016/s0966-6362(01)00156-4 12127181

